# Carrot flour-enriched pasta with *in vitro* antioxidant activity and capacity of modulating inflammatory pathways in human colon cell model

**DOI:** 10.3389/fnut.2026.1818508

**Published:** 2026-05-20

**Authors:** Alessia Lisi, Giusy Rita Caponio, Maria Calasso, Grazia Tamma, Ilenia Ceglie, Leonardo Mancini, Graziana Difonzo, Fabio Minervini, Maria De Angelis

**Affiliations:** 1 Department of Soil, Plant and Food Sciences, University of Bari Aldo Moro, Bari, Italy; 2 Department of Bioscience, Biotechnology and Environment, University of Bari Aldo Moro, Bari, Italy

**Keywords:** anti-inflammatory activity, antioxidant activity, beta-carotene, carrot flour, dietary fibre, enriched pasta

## Abstract

Wheat-based pasta, a staple food for many populations, represents a potential vehicle for bioactive compounds and micronutrients. This study aimed to enhance the nutritional and functional properties of fresh pasta by incorporating 12.5% orange or purple carrot flour. Pasta containing orange (O) or purple (P) carrot flour and a conventional pasta (control) were characterized in terms of nutritional composition, sensory profile and microbiological stability. The antioxidant and anti-inflammatory properties were investigated using *in vitro* assays with the human Caco-2 cell line. Both enriched pastas could be labelled as “rich in fibre,” while only pasta O qualified as a “source of vitamin A.” The enriched samples exhibited increased antioxidant capacity, attributable to the high content of phenolic compounds and anthocyanins in P and β-carotene in O. Exposure of Caco-2 cell to pasta digests in the presence of a synthetic pro-oxidant did not result in significant differences in reactive oxygen species levels. However, P digest, after colonic fermentation, induced a decrease of NF-κB phosphorylation, suggesting an inhibitory effect on the pro-inflammatory response. Both enriched pastas led to a decrease in vimentin expression, while an increase in E-cadherin expression was exclusively observed in cells treated with the P digest, as confirmed by immunofluorescence analysis. Microbiologically, all pasta samples were stable for 110 days. Overall, using carrot flour, particularly from purple cultivars, has proved to be an effective strategy of improving the nutritional and functional properties of fresh pasta while maintaining good sensory profile.

## 1 Introduction

Carrot (*Daucus carota* L.), a biennial plant from the *Apiaceae* family, is the second most widely consumed vegetable worldwide after potato. It is widely appreciated for its crunchy texture, refreshing flavour, and rich nutritional profile (1). Carrot provides a broad range of nutrients and health-promoting phytochemicals, including dietary fibre, carotenoids, anthocyanins, vitamins, minerals and other bioactive compounds, with carotenoids representing both potent antioxidants and precursor of vitamin A (retinol) (2). During industrial processing such as peeling and slicing, carrots generate 30–40% of by-products/waste, which still contain substantial levels of carotenoids and other phytochemicals (3, 4). Although these residues are commonly used for composting or animal feed, the growing interest in sustainable valorisation has encouraged their use for producing value-added ingredients, including carrot powder rich in β-carotene and phenolics (5, 6). Pasta is an interesting vehicle for such ingredients, as its reformulation is increasingly explored to enhance nutritional value and provide functional benefits (7, 8). The demand for enriched pasta – higher in essential amino acids, minerals (9, 10), fibre and vitamins (11)– continues to grow. The global pasta market is expected to reach a value of 99.24 billion USD by 2030 (7), reflecting the growing demand for healthier products. Consumers are increasingly willing to pay higher prices for products offering added beneficial characteristics, such as whole grain, legume-based, low-calorie and gluten-free options (7). However, the use of alternative ingredients can affect the sensory quality of the final product (8). Therefore, ongoing research aims to achieve an optimal balance between nutritional enhancement and desirable sensory attributes. Previous studies have shown that incorporating carrot powder can improve the nutritional composition, organoleptic characteristics, and shelf life of pasta, resulting in an enriched pasta with higher content of fibre, minerals (calcium, iron, potassium and sodium), vitamins (B1, B3, B6, C, E and K) and β-carotene (12, 13). Use of 1–3% carrot flour, combined with relatively low drying temperatures (50 °C), increased nutritional value of dried pasta without compromising its technological properties (14). As reported by Porto dalla Costa *et al*. (2016), enrichment of pasta with 20% of flour obtained from minimally processed carrot residues increased its nutritional value, functional properties, and antioxidant capacity (15). Similarly, Gastélum-Estrada et al. (16) reported notable antioxidant and anti-inflammatory effects of carrot-based juices, thanks to their high content in some phenolic compounds. However, research remains limited, particularly regarding the use of different carrot cultivars and the assessment of potential health effects in human intestinal cell models. Indeed, both the concentration and profile of bioactive compounds differ substantially among carrot genotypes (5). Orange carrot cultivars are characterized by high levels of carotenoids and the resulting lipophilic antioxidant potential. Cultivars with darker colours (e.g., purple) are distinguished by high content of phenolic acids (e.g., chlorogenic, caffeic, ferulic, p-coumaric) and anthocyanins (5). This study aimed to characterize industrially produced fresh pasta enriched with flour obtained from orange or purple carrot cultivars, compared with conventional pasta. Besides chemical, microbiological, colour, sensory, and nutritional analyses, the potential health implications of enriched pasta were investigated using human carcinoma Caco-2 cell line.

## 2 Materials and methods

### 2.1 Raw materials and reagents

Semolina (milled grains of *Triticum turgidum* subsp. *durum*, with particles size ranging from 180 to 355 μm) was supplied by Industria Molitoria F.lli Martimucci S.r.l. (Altamura, Italy), and its gross composition was as follows: moisture 15%, carbohydrates 75% dry weight (dw), fibre 3% dw, proteins 13.3% dw, dry gluten 11% dw, lipids 1.6% dw, and ash 0.75% dw. Two types of carrot flours (particles size smaller than 1 mm, moisture below 10%, carbohydrates 33.3–39.39% dw, fibre 44.6–46.1% dw, proteins 4.2–9.4% dw, lipids 1.7–2.37% dw) were provided by Aureli Mario SS Agricola: from roots of either orange (*Daucus carota* var. *sativus*, “Carota dell’Altopiano del Fucino PGI”) or purple carrots (*D. carota* var. *atrorubens*). *α*-Amylase, calcium chloride, pepsin, hydrochloric acid, pancreatin from porcine pancreas, bile salts, sodium bicarbonate, sodium carbonate, sodium hydroxide, 2,2-diphenyl-1-picrylhydrazyl (DPPH∙), Folin–Ciocalteu reagent, gallic acid, Trizma hydrochloride (Tris–HCl), dihydrorhodamine-123, Triton X-100, sodium deoxycholate, sodium dodecyl sulfate (SDS), ethylenediaminetetraacetic acid (EDTA), tert-butyl hydroperoxide (tBHP), 5-sulfosalicylic acid, amino acid analytical standard (HPLC grade), tryptophan (Trp, HPLC grade), ornithine (Orn, HPLC grade), *γ*-aminobutyric acid (GABA, HPLC grade), paraformaldehyde, peptone, bovine serum albumin (BSA), as well as the protease and phosphatase inhibitors phenylmethylsulfonyl fluoride (PMSF), leupeptin, pepstatin, sodium fluoride, and sodium orthovanadate, were purchased from Sigma-Aldrich (Milan, Italy). Cyanidin-3-O-glucoside was purchased from Phytoplan (Heidelberg, Germany). Calcein-AM (UltraPure Grade), Alexa Fluor™ 488–conjugated antibodies, MEM Non-Essential Amino Acids Solution (100×), Dulbecco’s Modified Eagle Medium (DMEM), and foetal bovine serum (FBS) were obtained from Thermo Fisher Scientific (Waltham, MA, USA). The ninhydrin reagent and the running buffers required for the analysis of free amino acids were supplied as part of a commercial kit provided by Biochrom Ltd. (Cambridge Science Park, UK). Unless otherwise specified, all reagents were of analytical grade.

### 2.2 Pasta preparation

Preliminary trials were performed at laboratory scale at the University of Bari Aldo Moro, to select the percentage of carrot flour (range 3–25%) that could be used as semolina substitute to obtain an enriched pasta that could be acceptable from a sensory point of view (17). The internal panel evaluated that 12.5% would be not detrimental to acceptability, while putatively increasing nutritional value of pasta, compared to conventional pasta, based only on water and semolina. Three types of pasta were then manufactured at the pilot plant (Pavan brand, GEA Group Aktiengesellschaft, Düsseldorf, Germany) of Industria Molitoria F.lli Martimucci S.r.l.: conventional (*alias* “control”) pasta; pasta enriched with orange (O) carrot flour; pasta enriched with purple (P) carrot flour. The recipe of control pasta was: 49.99 kg of semolina mixed with 15 L of water; the recipe for O and P was: 43.75 kg of semolina mixed with 6.25 kg of carrot flour (12.5% substitution) and 20 L of water. After kneading dough for 20 min, pasta was extruded through Teflon draw-plate. Fresh pasta underwent thermal treatment (73 °C; 3 min), was packaged in modified atmosphere (N_2_ 70%, CO_2_ 30%), heat-treated again (73 °C; 3 min) and stored at 4 °C for 110 days.

### 2.3 Nutritional characterization of raw pasta

Nutritional characterization was performed on raw (uncooked) samples of control, O, and P pastas by the external laboratory Bonassisa Lab S.p. A. (Foggia, Italy). The analytical methods were: energy value (MI 02.067 rev.052023), moisture (ISTISAN Reports 1996/34 p.7 – Method B), ash content (ISTISAN Reports 1996/34 p.77), nitrogenous substances (N × 6.25) (MI 02.310 rev.022022), carbohydrates (calculated with fibre fraction deduction) (MI 02.705 rev.042023), fructose, glucose, sucrose, maltose, lactose, (MI 02.884 rev00 2022), total soluble sugars (MI 02.884 rev.002022), total dietary fibre (AOAC 985.291986), soluble fibre (AOAC 991.431994), insoluble fibre (AOAC 991.431994), total lipids content (acid hydrolysis method) (ISTISAN Reports 1996/34 p.41– Method A), palmitic acid (C16:0), stearic acid (C18:0), oleic acid (C18:1), linoleic acid (omega-6) (C18:2), *α*-linolenic acid (omega-3) (C18:3) (MI 02.733 rev.062023), sum of saturated fatty acids, sum of monounsaturated fatty acids, sum of polyunsaturated fatty acids (MI 02.733 rev.062023), vitamin A (as β-carotene) (UNI EN 12823–2:2000), vitamin C (ISTISAN Reports 1996/34 p.157), vitamin E (as *α*-tocopherol equivalent) (ISTISAN Reports 1996/34 p.137), sodium, calcium, nickel, magnesium, potassium, iron, zinc, and phosphorus (MI 02.185 rev.032024).

### 2.4 pH, Total Titratable Acidity, water activity, and microbiological analyses of raw pasta

pH and Total Titratable Acidity (TTA) were determined after homogenization (3 min treatment) of 10 g of pasta with 90 mL of distilled water in a lab blender (Bag Mixer, Interscience International, Roubaix, France). The pH was determined by means of a pH-meter with a food-penetrating probe (Model 507, Crison, Milan, Italy). TTA was expressed as the volume (mL) of 0.1 M NaOH solution required to adjust the pH of the homogenized suspension to 8.3. Water activity (A_w_) was measured at 25 °C using the Acqualab 4TE (Decagon Devices Inc., USA) (18). Before measurement, raw pasta samples were coarsely ground to obtain representative portions and placed in the disposable sample trays (4 cm diameter) provided with the instrument. The trays were filled to completely cover the flat surface, up to approximately half its height, avoiding compaction of the material.

On the day of production (T0), as well as after 30 (T30), 60 (T60), 90 (T90), and 110 (T110) days from production, pasta samples were subjected to microbiological analyses, to enumerate the following microbial groups (the reference method used is indicated in brackets): total aerobic mesophilic bacteria (TMA) (UNI EN ISO 4833-1:2013), mesophilic lactic acid bacteria (LAB) (ISO 15214:1998), *Enterobacteriaceae* (ISO 21528-2:2017), enterococci (EN ISO 11133:2014), coagulase-positive staphylococci (UNI EN ISO 6888-2:2004), yeasts and moulds (ISO 21527-2:2008). Prior to inoculation of media in 90 mm-diameter Petri dishes, 25 g of raw pasta were homogenized (2 min in a lab blender) with 225 mL of 0.1% Buffered Peptone Water and serially diluted (19).

### 2.5 Free amino acids profile of pasta before and after cooking

The profile of total free amino acids (FAA) was assessed on aqueous extracts of raw pasta (uncooked, U) and cooked (C) pasta. Aliquots of raw and cooked pasta were preliminary freeze-dried (Lyostar II, FTS kinetics, Stone Ridge, NY), then finely ground using a mortar. An amount of dried sample corresponding to 1 g of fresh weight was mixed with 4 mL of 50 mM Tris–HCl (pH 8.8); after incubation for 1 h at 4 °C, under gentle stirring (150 rpm in orbital shaker ARGOlab SKO-D XL, Giorgio Bormac SRL, Carpi, MO, Italy), the suspension was centrifuged (15,000 g, 20 min at room temperature). Cold solid 5-sulphosalicylic acid was added to the resulting supernatant (final concentration 5%, v/v) to precipitate proteins and peptides. After incubation (1 h at 4 °C), samples were centrifuged again (15,000 g, 15 min at room temperature). The final supernatant was filtered through a 0.22 μm cellulose acetate non-sterile syringe filter and stored at −20 °C until analysis. Total and individual FAA analysis was performed through chromatographic separation using a Biochrom 30 + Automatic Amino Acid Analyser (Biochrom) equipped with a Li-cation-exchange column (Ultropac™ Li, 20 cm × 0.46 cm). Amino acids were separated using a stepwise elution program with six lithium citrate buffers at increasing pH and column temperature, according to the manufacturer’s high-performance method. The flow rate of the mobile phase was set at 31 mL h^−1^. The gradient used was as follows: 35 s, Buffer 1 (pH 2.80) at 26 °C, followed by 26 min of Buffer 2 (pH 3.00) at 26 °C. Intermediate-eluting amino acids were separated using Buffer 3 (pH 3.15), at 43 °C for 15 min, followed by Buffer 4 (pH 3.5) at 69 °C for 21 min. Late-eluting amino acids were resolved using Buffer 5 (pH 3.55) at 81 °C, while Buffer 6, a regeneration buffer containing lithium hydroxide monohydrate (1.3%, w/v), was applied at 81 °C. The system was finally re-equilibrated with Buffer 1 prior to the subsequent analysis. After chromatographic separation, amino acids were post-column derivatized with ninhydrin, delivered at a constant flow rate of 25 mL h^−1^. The derivatization reaction took place in a reaction coil maintained at 135 °C. Detection was performed by measuring absorbance at 570 nm for primary amino acids and 440 nm for amino acids (e.g., proline). Quantitative analysis was performed using an external standard consisting of a mixture of amino acids (AAS18, Supelco, Sigma-Aldrich), supplemented with Trp, Orn, and GABA, all at known concentrations, namely 1.25 μmol of 0.1 N HCl for L-cystine and 2.5 μmol mL^−1^ for all the other amino acids (20). Amino acids were identified by comparing their retention times with those of the corresponding standards.

### 2.6 Colour and sensory analysis of pasta

Brightness (L*), red index (a*), yellow index (b*), and total colour difference (ΔE) were determined on raw and cooked pasta using the colorimeter Chromameter CM-600d (Konica Minolta Sensing, Osaka, Japan). The ΔE index indicates the chromaticity coordinates against the instrument’s blank (21). Sensory evaluation was conducted following the ethical guidelines in the Laboratory of Food Microbiology of the Department of Plant, Soil, and Food Science at the University of Bari Aldo Moro, Italy. The evaluation was carried out following the UNI EN ISO guidelines 13,299 and involved 10 semi-trained judges. Panellists were informed about the study’s objectives and provided written consent to participate in the sensory analysis, in line with the ethical guidelines of the laboratory. Pre-test sessions were held to establish a list of descriptors and to assess the judges’ ability to discriminate, as well as their consistency and reliability. The samples were randomized and presented (5 g for each type) to the panellists in white dishes marked with alphanumeric codes. Panellists were instructed to cleanse their palates with water between tasting different samples. The following sensory attributes were considered: colour intensity and appearance (visual evaluation); bitterness, sourness, typical carrot flavour, presence of particles, and persistence of flavour (taste and sensations during chewing and swallowing); typical carrot odour and off odour. Each attribute was evaluated using a 10-point structured scale from 0 to 9 (22, 23).

### 2.7 *In vitro* pasta digestion and simulated colonic fermentation

The pasta underwent the *in vitro* digestion as previously described (24). Briefly, 10 g of cooked pasta were added with 50 mL of distilled water and homogenized for 2 min in a lab blender. The following enzyme solutions were added to the suspension: *α*-amylase (20 mg in 6.25 mL 1 mM CaCl_2_) to simulate oral digestion, pepsin (2.7 g in 25 mL of 0.1 M HCl, pH 2) to simulate gastric digestion, pancreatin (560 mg) and bile salt (3.5 g in 125 mL of 0.1 M NaHCO_3_, pH 7) to simulate intestinal digestion. The digested pasta samples were stored at −20 °C until analyses. To obtain the faecal microbiota inoculum, a fresh faecal sample (<1 h from delivery) from a healthy volunteer, a member of the research group who signed the informed consent and did not take drugs or probiotics in the last three months before the sample collection, was added at 32% (w/v) to saline solution (NaCl 0.9% w/v) and homogenized. The resulting faecal slurry was added at 20% (v/v) (25) to faecal medium that had been prepared as described previously (26). The faecal batch included 7.5 mL of faecal medium, 0.5 g of pasta digested as detailed above, and 2 mL of faecal slurry. Samples were incubated anaerobically (42 h, 37 °C) under stirring (100 g). Then, aliquots (5 mL) from faecal batches, mixed with 45 mL of sterilized saline solution, were diluted and subjected to plate counts, to estimate different microbial groups with the agar media and incubation conditions reported in the Supplementary Table S1. All the media were purchased from Oxoid Ltd. (Basingstoke, Hampshire, UK), except for *Bifidobacterium* agar, which was purchased from Becton Dickinson (Le Pont de Claix, SA, France).

### 2.8 Antioxidant activity of pasta before and after cooking

The antioxidant activity was estimated as the scavenging activity of the ethanolic extracts of pasta against DPPH∙ as reported by Caponio et al. (27) with modifications reported by Limongelli et al. (28). Specifically, 10 mL of an ethanol:water solution (80:20, v/v) was added to 1 g of pasta. The mixture underwent stirring (using a VM4 IDL stirrer) for 10 min at room temperature, followed by treatment in an ultrasonic bath (Ultrasound CP104, CEIA, Civitella di Val di Chiana, AR, Italy) for 15 min and centrifugation (MOD SL 16 R Centrifuge, Thermo Fisher Scientific, MA, USA) at 13,552 g for 10 min at 25 °C. The resulting supernatant was collected, and the pellet underwent two additional extractions under the same conditions as mentioned earlier. The supernatants from all three extractions were combined and subjected to determination of antioxidant activity. The free radical scavenging activity against DPPH∙ was measured as described by Caponio et al. (27) with slight modifications. Briefly, 50 μL of each sample were added to 950 μL of DPPH∙ solution (0.0031 g/100 mL in ethanol). Blank and positive control, containing 50 μL of ethanol-water solution or the synthetic antioxidant butylated hydroxytoluene (BHT) as reference (1 g L^−1^ in ethanol-water solution), respectively, instead of sample, were included in the analysis. After incubation (in dark, 30 min at 25 °C), the absorbance was measured at 517 nm. The free radical scavenging activity was calculated as follows:

$$\text{DPPH}\cdot \text{scavenging activity}\;(\%)=[\frac{\left(\begin{array}{l} A\;\text{blank}\\ -A_{\text{sample}} \end{array}\right)}{A\;\text{blank}}]\times 100$$

### 2.9 Total phenolic compounds

Phenolic compounds were extracted from semolina/carrot flours and pasta according to Difonzo et al. (29) and Troilo et al. (30), with slight modifications. Ten millilitres of a methanol/water mixture (80:20, v/v) was added to 1 g of semolina/carrot flour or pasta (uncooked and cooked). After shaking for 10 min, the mixtures were sonicated in an ultrasound bath, operating at 39 kHz and 200 W, for 15 min at room temperature, followed by shaking for 30 min. The suspensions were then centrifuged at 12,000 g for 15 min at 4 °C, and the supernatants were filtered through nylon filters (0.45 μm pore size, Sigma, Ireland). The extracts were used for subsequent analyses. Total phenolic content (TPC) was determined using the Folin–Ciocalteu method according to Difonzo et al. (31), with slight modifications. Briefly, 20 μL of filtered extract was mixed with 980 μL of deionized water and 100 μL of Folin–Ciocalteu reagent. After 3 min, 800 μL of 7.5% sodium carbonate was added, and the mixture was incubated at room temperature for 60 min. Absorbance was measured at 720 nm using a Cary 60 spectrophotometer (Agilent Technologies, Cernusco sul Naviglio, Milan, Italy). TPC was obtained by performing a calibration curve with gallic acid (y = 0.0052x + 0.0029; R^2^ = 0.9991). The results were expressed as μg gallic acid equivalents (GAE) g^−1^ (31).

### 2.10 Anthocyanins

Anthocyanins were extracted from semolina/carrot flours and pasta (both uncooked and cooked) following Troilo et al. (30), with minor modifications. Approximately 1 g of sample was mixed with 10 mL of methanol/water/formic acid (80:18:2, v/v/v), subjected to sonication for 10 min, and shaken for 30 min. The extracts were then centrifuged at 12,000 g for 10 min at 4 °C. The supernatants were collected, while the pellets were re-extracted with the same solvent three times. Total anthocyanin content was determined by UV–vis spectrophotometry according to Pasqualone et al. (32). Filtered extracts (0.45 μm nylon filter) were analysed at 535 nm using a Cary 60 UV–vis spectrophotometer. Quantification was performed using the external standard method, based on a calibration curve prepared with cyanidin-3-O-glucoside: y = 0.0352x + 0.0067(R^2^ = 0.9983). Results were expressed as μg cyanidin-3-O-glucoside g^−1^.

### 2.11 Functional evaluation of digested pasta samples on cell cultures

#### 2.11.1 Cell cultures and treatments

The human colorectal adenocarcinoma cell line Caco-2 (supplied by ATCC, HTB-37™) was maintained at 37 °C, 95% air, 5% CO_2_ in DMEM supplemented with foetal bovine serum (10% v/v), non-essential amino acids (1% v/v), 100 U mL^−1^ penicillin and 100 μg mL^−1^ streptomycin (33). The pasta samples that had been previously digested and subjected to colonic fermentation (described in paragraph 2.7), were neutralized to pH 6.9 using 35% NaOH, then centrifuged (18,500 g, 20 min). To eliminate the residual turbidity, the supernatant was filtered through 0.45 μm cellulose acetate membranes and stored at −80 °C until further analysis. For treatments, cells were left under basal condition (untreated, CTR-) or treated with extracts of digested pasta for 24 h.

#### 2.11.2 Antibodies

Nuclear Factor kappa B (NFκB) (1:200) and NFκB-pS536 (1:200) were acquired by Santa Cruz Biotechnology (Santa Cruz, CA, USA). Vimentin (1:2500) was obtained from ProteinTech Group (Rosemont, IL, USA), and E-cadherin (1:800) was obtained from Millipore Merck (Merck KGaA, Darmstadt, Germany). The Anti-Rabbit IgG (whole molecule) and the Anti-Mouse IgG (whole molecule) secondary antibodies were obtained from Sigma-Aldrich.

#### 2.11.3 Calcein-AM cell viability assay

To evaluate the cell viability, cells were exposed for 24 h to digested pasta extracts at increasing concentration series (1:200; 1:150; 1:100; 1:50). After treatments, cells were incubated with calcein-AM (1 μM) at 37 °C for 45 min, and then the fluorescence signal was measured as previously described (34).

#### 2.11.4 Reactive Oxigen Species (ROS) detection

ROS were detected as described elsewhere (35). Briefly, cells were exposed for 24 h to digested pasta extracts. After treatments, cells were incubated with 10 μM dihydrorhodamine-123 at 37 °C for 30 min. As a positive control, cells were treated with 2 mM tBHP supplemented in cultured medium, also in combination with digested pasta extracts. Then cells were incubated at 37 °C for 30 min. Cells were subsequently lysed in RIPA buffer containing 150 mM NaCl, 10 mM Tris–HCl pH 7.2, 3.5 mM SDS, 1.0% Triton X-100, 24 mM sodium deoxycholate, and 5 mM EDTA. Lysates were centrifuged (12,000 g, 10 min, 4 °C), and the resulting supernatants were used for ROS detection. The fluorescence emission signals were recorded using a FLUOstar Omega fluorometer (5.10 R2, BMG LABTECH, Offenburg, Germany) at excitation and emission wavelengths of 508 and 529 nm, respectively.

#### 2.11.5 Cell lysates and Western blotting

All the equipments and softwares used for Western blotting were purchased from Bio-Rad Laboratories Inc. (Hercules, CA, USA). Caco-2 cells were plated onto 60 mm diameter Petri dishes, treated with digested pasta extracts, and then lysed in RIPA buffer containing 1 mM PMSF, 2 mg mL^−1^ leupeptin, 2 mg mL^−1^ pepstatin, 10 mM sodium fluoride, and 1 mM sodium orthovanadate. The supernatants from lysed Caco-2 cells were subjected to Western blot analysis. Proteins were separated using 10% or 12% stain-free polyacrylamide gels. Subsequently, protein bands were electrophoretically transferred onto Immobilon-P PVDF membranes and incubated with EveryBlot blocking solution (Bio-Rad Laboratories). Blots were then incubated at 4 °C overnight with primary antibodies. To detect immunoreactive bands, horseradish peroxidase-conjugated secondary antibodies were used. Membranes were then treated with Clarity™ Western ECL substrate, and signals were visualized using the ChemiDoc imaging system. The resulting bands were normalized to total protein using stain-free gel technology. Densitometric analysis was performed using Image Lab software.

#### 2.11.6 Immunofluorescence

Caco-2 cells were seeded on 12 mm diameter glass coverslips and treated with digested pasta extracts for 24 h. Cells were processed by immunofluorescence protocol, as described by Angelini et al. (36). Briefly, the cells were fixed by immersion in 4% (w/v) paraformaldehyde solution for 20 min, washed three times with phosphate-buffered saline (PBS) with calcium and magnesium, and subsequently permeabilized with 0.1% Triton X-100 in PBS for 5 min. The unspecific binding sites were blocked by incubation with 1% BSA in PBS. Then, the vimentin and E-cadherin specific primary antibodies were incubated overnight at 4 °C, followed by the incubation (1 h at 37 °C) with the 488 Alexa Fluor-conjugated secondary antibodies (Thermo Fisher Scientific). After washes, glass coverslips were mounted onto glass slides with Mowiol® mounting medium. Images were acquired with a confocal laser-scanning fluorescence microscope Leica TCS SP2 (Leica Microsystems, Heerbrugg, Switzerland) using identical acquisition settings.

### 2.12 Statistical analysis

Two replicate batches of pasta were manufactured on the same day. All analyses were performed in triplicate. Significant (*p* ≤ 0.05) differences were determined by One-way Analysis of Variance (ANOVA), followed by Tukey’s test for multiple comparisons. Experiments on human colon cancer cells were performed in two independent biological replicates, each consisting of separate cell cultures, and that each biological replicate was analysed with three technical replicates. The variability of the data is presented as mean ± SEM, and statistical significance was assessed using One-way ANOVA followed by Tukey’s test. The statistical analysis was carried out using the software GraphPad Prism 10.3.1 (GraphPad Software, Boston, MA, USA).

## 3 Results

### 3.1 Nutritional composition of pasta enriched with carrot flour

Both pasta enriched with orange carrot flour (O) and pasta enriched with purple carrot flour (P) had lower energy values and carbohydrates than conventional pasta (control) (Table 1). Fructose, glucose, and lactose were below the detection limit (0.5%). The enriched pastas were characterized by a higher concentration of total, soluble, and insoluble fibre than the control. While vitamins C and E were below the detection limit (1 mg/100 g) in any type of pasta, β-carotene (vitamin A) was found at the highest (*p* ≤ 0.05) concentration in O. As regards minerals, both pastas enriched with carrot flour had higher (*p* ≤ 0.05) concentrations of sodium, calcium, and especially potassium (around 4 g kg^−1^) than the control. The latter was characterized by the highest (*p* ≤ 0.05) concentration of the micronutrient zinc, whereas no significant differences were found for magnesium, phosphorous, iron, and nickel (Table 1). Other important nutritional contributions could be represented by individual FAA. Both pastas enriched with carrot flour showed higher (*p* ≤ 0.05) contents of asparagine and alanine than the control (Supplementary Table S2). In addition, O was characterized by higher contents of Trp, valine, and GABA, and P showed higher contents in aspartic acid, glutamic acid, and especially arginine. After cooking, we found an overall decrease of FAA, but both the enriched pastas still had higher contents of the abovementioned amino acids than conventional pasta (Supplementary Table S2).

**Table 1:** Nutritional characterization of conventional pasta (control), pasta enriched with orange (O) and purple (P) carrot flour.

Nutritional facts	Control	O	P
Energy value (kcal/100 g)	283.00 ± 3.61^a^	256.00 ± 4.58^b^	238.33 ± 3.79^c^
Energy value (kJ/100 g)	1199.67 ± 14.84^a^	1082.7 ± 19.55^b^	1006.67 ± 15.5^c^
Moisture (%)	28.82 ± 0.88^c^	32.7 ± 1.18^b^	37.40 ± 0.86^a^
Ash (%)	0.82 ± 0.02^c^	1.20 ± 0.02^a^	1.21 ± 0.01^b^
Nitrogenous substances (Nx6.25) (%)	14.26 ± 0.23^a^	12.57 ± 0.75^b^	13.31 ± 0.21^b^
Carbohydrates (with fibre fraction deduction) (%)	79.29 ± 0.35^a^	73.28 ± 0.86^b^	71.94 ± 0.47^c^
Sucrose (%)	<0.50^b^	2.10 ± 0.27^a^	2.01 ± 0.02^a^
Maltose (%)	1.64 ± 0.03^a^	2.17 ± 0.76^a^	2.34 ± 0.08^a^
Total soluble sugars (%)	1.64 ± 0.03^b^	4.25 ± 1.01^a^	4.36 ± 0.10^a^
Total dietary fibre (g/100 g)	3.93 ± 0.09^c^	11.34 ± 0.19^b^	11.82 ± 0.30^a^
Soluble fibre (g/100 g)	0.89 ± 0.20^c^	3.01 ± 0.03^b^	3.52 ± 0.19^a^
Insoluble fibre (g/100 g)	3.04 ± 0.24^c^	8.34 ± 0.18^a^	8.31 ± 0.18^a^
Total lipids (acid hydrolysis method)	1.73 ± 0.06^a^	1.63 ± 0.03^b^	1.76 ± 0.02^a^
Palmitic acid (C16:0) (%)	25.91 ± 0.67^c^	28.71 ± 0.64^b^	30.40 ± 0.73^a^
Stearic acid (C18:0) (%)	2.42 ± 0.10^a^	3.19 ± 0.04^a^	3.39 ± 0.78^a^
Oleic acid (C18:1) (%)	24.99 ± 0.14^a^	27.41 ± 1.48^a^	28.06 ± 0.77^a^
Linoleic acid (C18:2) (omega-6) (%)	82.13 ± 0.99^b^	84.32 ± 0.87^b^	91.87 ± 2.44^a^
Alpha-linolenic acid (C18:3) (omega-3) (%)	5.06 ± 0.29^a^	4.97 ± 0.15^a^	6.05 ± 0.50^a^
Sum of saturated fatty acids (%)	28.33 ± 0.74^c^	31.90 ± 0.63^b^	33.79 ± 0.42^a^
Sum of monounsaturated fatty acids (%)	24.99 ± 0.14^b^	27.41 ± 1.48^a^	28.06 ± 0.77^a^
Sum of polyunsaturated fatty acids (%)	87.19 ± 1.19^b^	89.29 ± 0.80^b^	97.92 ± 2.05^a^
Vitamin A (as b-carotene) (μg(RE)/100 g)	3.11 ± 0.18^b^	209.06 ± 13.83^a^	3.68 ± 0.61^b^
Sodium (mg/kg)	21.55 ± 0.97^b^	623.54 ± 89.80^a^	649.07 ± 15.30^a^
Calcium (g/kg)	0.22 ± 0.05^c^	0.92 ± 0.15^b^	1.13 ± 0.03^a^
Nickel (mg/kg)	0.04 ± 0.0^a^	0.09 ± 0.04^a^	0.10 ± 0.00^a^
Magnesium (mg/kg)	342.84 ± 5.57^a^	424.01 ± 137.14^a^	459.83 ± 111.47^a^
Potassium (g/kg)	2.29 ± 0.08^c^	3.78 ± 0.46^b^	4.35 ± 0.14^a^
Iron (mg/kg)	11.62 ± 0.37^a^	12.70 ± 2.15^a^	14.22 ± 0.22^a^
Zinc (mg/kg)	12.83 ± 0.26^a^	11.84 ± 1.24^a^	12.86 ± 0.30^a^
Phosphorus (g/kg)	1.86 ± 0.06^a^	1.95 ± 0.48^a^	1.65 ± 0.04^b^

Data are expressed as mean ± SD. ^a–c^Within each column, values with different superscript letters were significantly different (*p* ≤ 0.05).

### 3.2 Intrinsic parameters and microbial loads of pasta

Water activity varied between 0.93 ± 0.05 (conventional pasta) and 0.96 ± 0.02 (pasta enriched with purple carrot flour, P), with no significant differences among the types of pasta subjected to this study (data not shown). The lowest (*p* ≤ 0.05) value of pH was found for P, whereas no differences were found between pasta enriched with orange carrot flour (O) and conventional pasta (control) (Table 2). Both pastas enriched with carrot flour showed the highest TTA, with no difference between them; however, P did not differ from the control, which showed the lowest (*p* ≤ 0.05) value of TTA. After cooking, an overall increase of pH (and, accordingly, a decrease of TTA) was found. P still had the lowest pH, not different from the control. No significant differences were found for TTA after cooking (Table 2). Microbiological analyses revealed that on the day of production (T0), TMA ranged from 1.8 (control) to 3.4 (P) log cfu g^−1^ (Supplementary Table S3). Presumptive LAB were detected at low cell density (1.6–2 log cfu g^−1^) just in the pasta enriched with carrot flour. Coagulase-positive staphylococci, *Enterobacteriaceae*, moulds and yeasts were below the detection limit. After 30 days of refrigerated storage (T30), TMA and LAB were found at cell density varying from 2.5 (control) to 3.7 (P), and from 2.1 (control) to 2.9 (O) log cfu g^−1^, respectively (Supplementary Table S3). At this stage of storage, staphylococci were detected, just in the control, at 2.3 log cfu g^−1^ (data not shown), whereas yeast cell density ranged between 2.3 (control) and 2.9 (P) log cfu g^−1^ (Supplementary Table S3). *Enterobacteriaceae* and moulds were not detected neither after 30 days nor after 110 days of storage. After 60 days (T60), TMA were the only detectable microbiological indicators (2.3–2.5 log cfu g^−1^). After 90 days (T90), besides TMA (2.6–3.2 log cfu g^−1^), we found LAB (2.4–2.6 log cfu g^−1^) just in the pasta enriched with carrot flour. At the end of shelf-life (T110), LAB, as well as staphylocci and yeasts, were below the detection limit, whereas TMA ranged from 2.9 (control) to 4.4 (O) log cfu g^−1^ (Supplementary Table S3).

**Table 2:** pH and Total Titratable Acidity (TTA) of conventional pasta (control), pasta enriched with orange (O) or purple (P) carrot flour, before (U) and after (C) cooking.

Pasta	pH	TTA (ml of NaOH 0.1 M)
Control-U	5.86 ± 0.06^b^	0.40 ± 0.07^bc^
O-U	5.97 ± 0.02a^b^	0.60 ± 0.02^a^
P-U	5.71 ± 0.01^c^	0.50 ± 0.04^ab^
Control-C	6.01 ± 0.01^a^	0.20 ± 0.00^c^
O-C	6.03 ± 0.04^a^	0.30 ± 0.07^c^
P-C	5.85 ± 0.04^b^	0.30 ± 0.03^c^

Data are expressed as mean ± SD. ^a–c^Within each column, values with different superscript letters were significantly different (*p* ≤ 0.05).

### 3.3 Effects of pasta enriched with carrot flour on colour and sensory quality

Colour analysis showed that, as expected, pasta enriched with purple carrot flour (P) had the lowest (*p* ≤ 0.05) values of brightness (L*) and yellow (b*) indexes, and the highest (*p* ≤ 0.05) value of red (a*) index (Table 3). Pasta enriched with orange carrot flour (O) did not significantly differ from the control, in terms of brightness, while being characterized by higher (*p* ≤ 0.05) values of a* index. It also showed the highest (*p* ≤ 0.05) value of b* index. The highest (*p* ≤ 0.05) colour differences, summarized by ΔE*, were found for P. Cooking did not affect any colour index (Table 3).

**Table 3:** Colour profile of conventional pasta (control), pasta enriched with orange (O) or purple (P) carrot flour, before (U) and after (C) cooking.

Pasta	L*	a*	b*	ΔE*
Control-U	84.55 ± 0.42^a^	−2.25 ± 0.19^c^	24.32 ± 1.42^b^	22.73 ± 1.05^c^
O-U	79.04 ± 0.89^a^	0.58 ± 0.34^b^	34.77 ± 2.5^a^	34.48 ± 2.65^b^
P-U	44.03 ± 3.70^b^	15.54 ± 1.60^a^	−1.21 ± 0.13^c^	53.51 ± 4.00^a^
Control-C	84.51 ± 0.27^a^	−2.39 ± 0.13^c^	22.73 ± 2.36^b^	21.86 ± 2.60^c^
O-C	76.59 ± 1.15^a^	−0.44 ± 0.09^b^	36.32 ± 0.66^a^	37.00 ± 1.14^b^
P-C	41.55 ± 3.92^b^	10.33 ± 0.65^a^	−1.27 ± 0.38^c^	54.62 ± 3.99^a^

Data are expressed as mean ± SD. ^a–c^Within each column, values with different superscript letters were significantly different (*p* ≤ 0.05).

**Figure 1:**
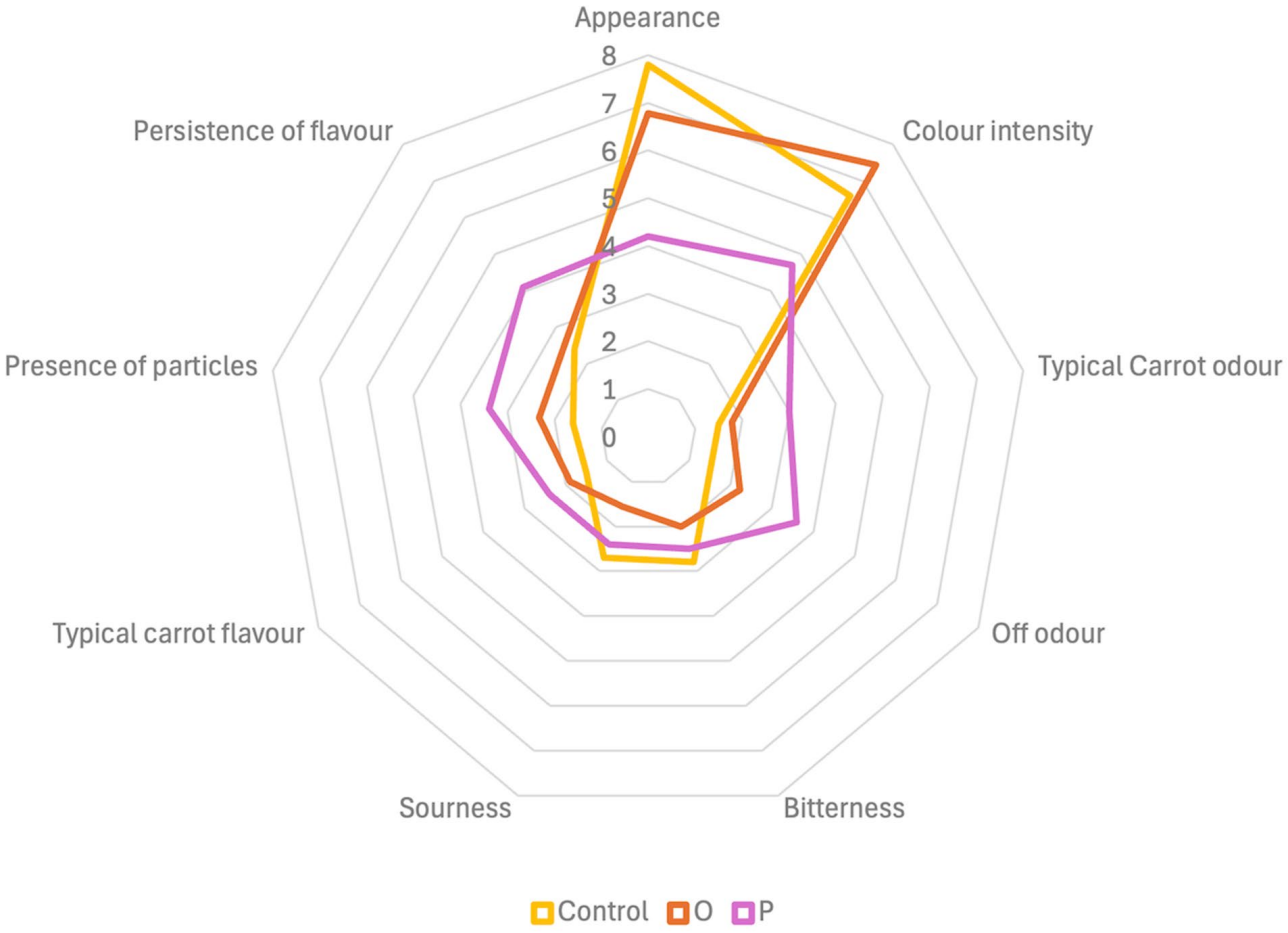
Spider web graph showing the results of the panel test carried out on conventional pasta (control), pasta enriched with orange (O) or purple (P) carrot flour.

Sensory analysis showed that some descriptors for the two-carrot flour-enriched pastas (O and P) were very different to those for conventional pasta (control) (Figure 1). Overall, the presence of particles slightly penalized the enriched pastas. However, O was appreciated for its intense orange hue, while P received lower scores due to colour loss and oxidation (7.84 ± 1.26 for O; 4.7 ± 1.42 for P). Off odours were low but more perceptible in O (2.2 ± 1.42) and P (3.6 ± 1.51) than in control (1.5 ± 1.08) (Figure 1). The enriched pastas showed lower bitterness (2.8 ± 1.03 for the control; 2.0 ± 1.05 for O; 2.5 ± 1.27 for P) and sourness (2.7 ± 1.25 for the control; 1.6 ± 0.70 for O; 2.5 ± 1.27 for P) than the control. Both the enriched pastas presented light carrot notes (both for the odour and flavour), considered a potential distinguishing element (Figure 1).

### 3.4 Effect of digested pasta on faecal microbiota

After 42 h of incubation of faecal extract with *in vitro* digested pasta, lower (*p* ≤ 0.05) cell densities of clostridia were found upon colonic fermentation of pasta with carrot flour, compared to control (Supplementary Table S4). In addition, presumptive LAB were found at lower (*p* ≤ 0.05) cell densities in the faecal extracts incubated with pasta enriched with purple carrot flour (P), than in those incubated with conventional pasta. No significant differences of cell densities were found for the other microbial groups (Supplementary Table S4).

### 3.5 *In vitro* antioxidant activity of pasta samples

**Figure 2:**
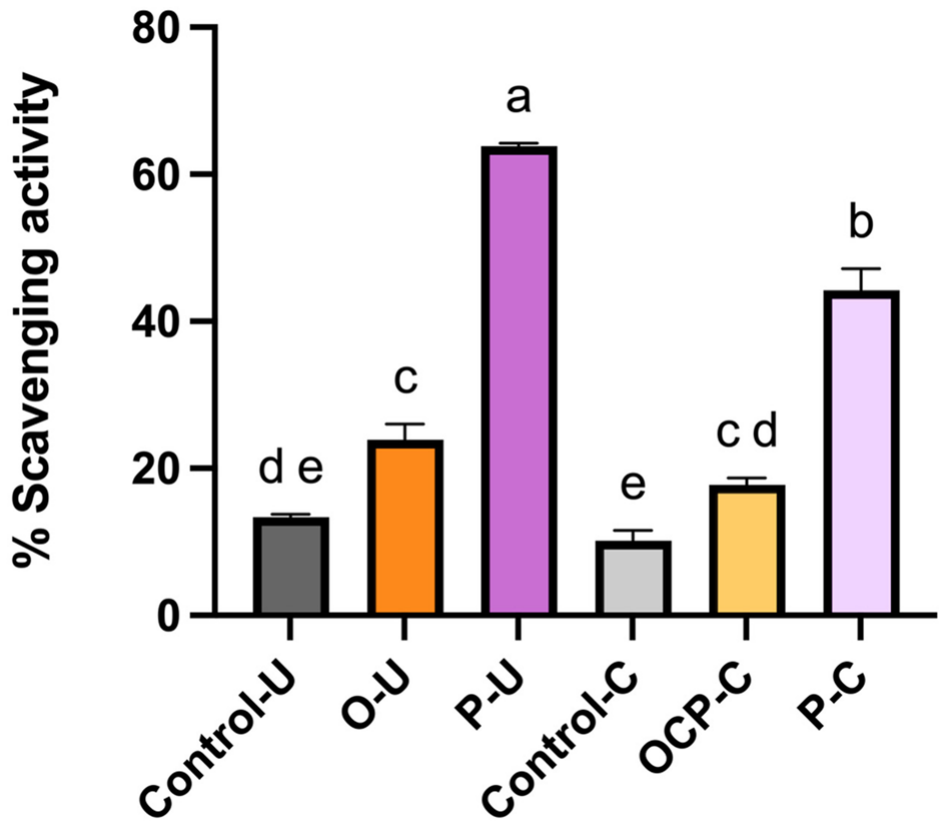
Radical scavenging activity of uncooked (U) or cooked (C) conventional pasta (Control) and enriched pasta orange (O) or purple (P) carrot flour. On the stacked bars, different letters denote a significant difference (*p* ≤ 0.05; one-way ANOVA).

Pasta enriched with purple carrot flour (P) showed the highest (*p* ≤ 0.05) radical scavenging activity, followed by orange carrot flour (O); on the contrary, control pasta had the lowest (*p* ≤ 0.05) *in vitro* antioxidant activity. After cooking, the antioxidant activity of P decreased, but was still significantly (*p* ≤ 0.05) higher than that of the control and of pasta enriched with orange carrot flour (O) (Figure 2).

### 3.6 Total phenols and anthocyanins of pasta and their link to raw materials

Pasta enriched with purple carrot flour (P) showed the highest (*p* ≤ 0.05) level of total phenols (Table 4). After cooking, total phenols decreased in all the pastas, but P was still characterized by the highest (*p* ≤ 0.05) value, three- to four-fold higher than the two other pastas. Anthocyanins characterized P too, although they decreased after cooking. They were detected only in trace amounts in the two other pastas (Table 4). These bioactive compounds originated mostly from purple carrot flour. Indeed, it contained the highest content of total phenols (3463.87 ± 19.98 μg GAE g^−1^), followed by orange carrot flour (1711.79 ± 13.75 μg GAE g^−1^), and semolina (1605.69 ± 12.61 μg GAE g^−1^). Anthocyanins were found at a concentration of 3214.62 ± 9.44 μg g^−1^ in purple carrot flour, whereas they were found in trace amounts in orange carrot flour and semolina.

**Table 4:** Concentrations of total phenols and anthocyanins in conventional pasta (control), pasta enriched with orange (O) or purple (P) carrot flour, before (U) and after (C) cooking.

Pasta	Total phenols (μg GAE/g)	Anthocyanins (μg/g)
Control-U	1108.23 ± 2.44^b^	Not detected
O-U	1033.18 ± 3.96^c^	Not detected
P-U	2055.36 ± 8.84^a^	286.92 ± 4.11^a^
Control-C	241.81 ± 5.26^e^	0.28 ± 0.00^c^
O-C	170.86 ± 3.32^f^	3.55 ± 0.01^c^
P-C	714.12 ± 0.03^d^	130.13 ± 0.54^b^

Data are expressed as mean ± SD. ^a–f^Within each column, values with different superscript letters were significantly different (*p* ≤ 0.05).

### 3.7 Biological characterization of digested pasta samples on Caco-2 cells

#### 3.7.1 Cell viability and ROS detection

The effect of increasing dilutions of digested pasta on cell viability is shown in Supplementary Table S5. At the lowest concentration tested (1:200 dilution), as well as at the 1:100 dilution, none of the samples affected cell viability, which remained comparable to CTR−. At a dilution of 1:150, O sample caused a significant (*p* ≤ 0.05) reduction of cell viability. At the highest concentration (1:50 dilution), both control and P pasta samples did not decrease cell viability, whereas the treatment with O sample resulted in significant (*p* ≤ 0.05) reduction in viability (Supplementary Table S5). Based on these results, subsequent experiments were carried out with digested pasta samples diluted 1:100.

**Figure 3:**
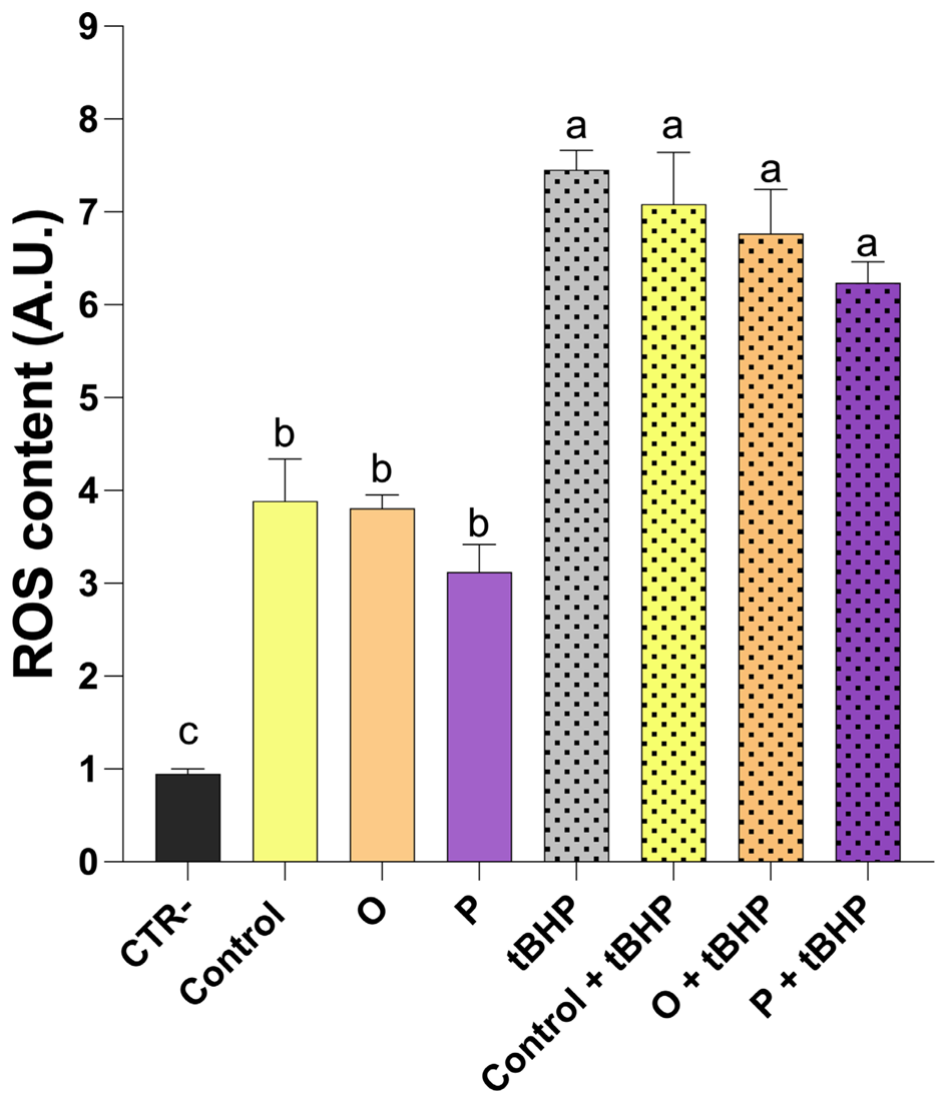
ROS content measured in Caco-2 cells treated for 24 h with diluted (1:100) digested pasta (CTR-, untreated cells; Control, conventional pasta; O, enriched pasta with orange carrot; P, enriched pasta with purple carrot) and exposed to the pro-oxidant tert-butylhydroperoxide (tBHP). Data are expressed as mean ± SEM. Bars with different letters indicate a significant difference (*p* ≤ 0.05).

To evaluate the antioxidant activity of the digested pasta extracts, the ROS levels of Caco-2 cells were measured. Treatment with all the digested pasta extracts resulted in an increased intracellular ROS level (Figure 3). As expected, in the presence of the pro-oxidant tBHP, the intracellular ROS levels increased. Although no statistically significant difference was found between cells exposed to tBHP and treated with digested pasta and those only exposed to tBHP, a downward trend of the ROS content was shown. In particular, cells treated with digested purple carrot pasta (P + tBHP) were characterized by a relatively low level of ROS (Figure 3).

#### 3.7.2 Effects on inflammation and epithelial–mesenchymal transition (EMT)

**Figure 4:**
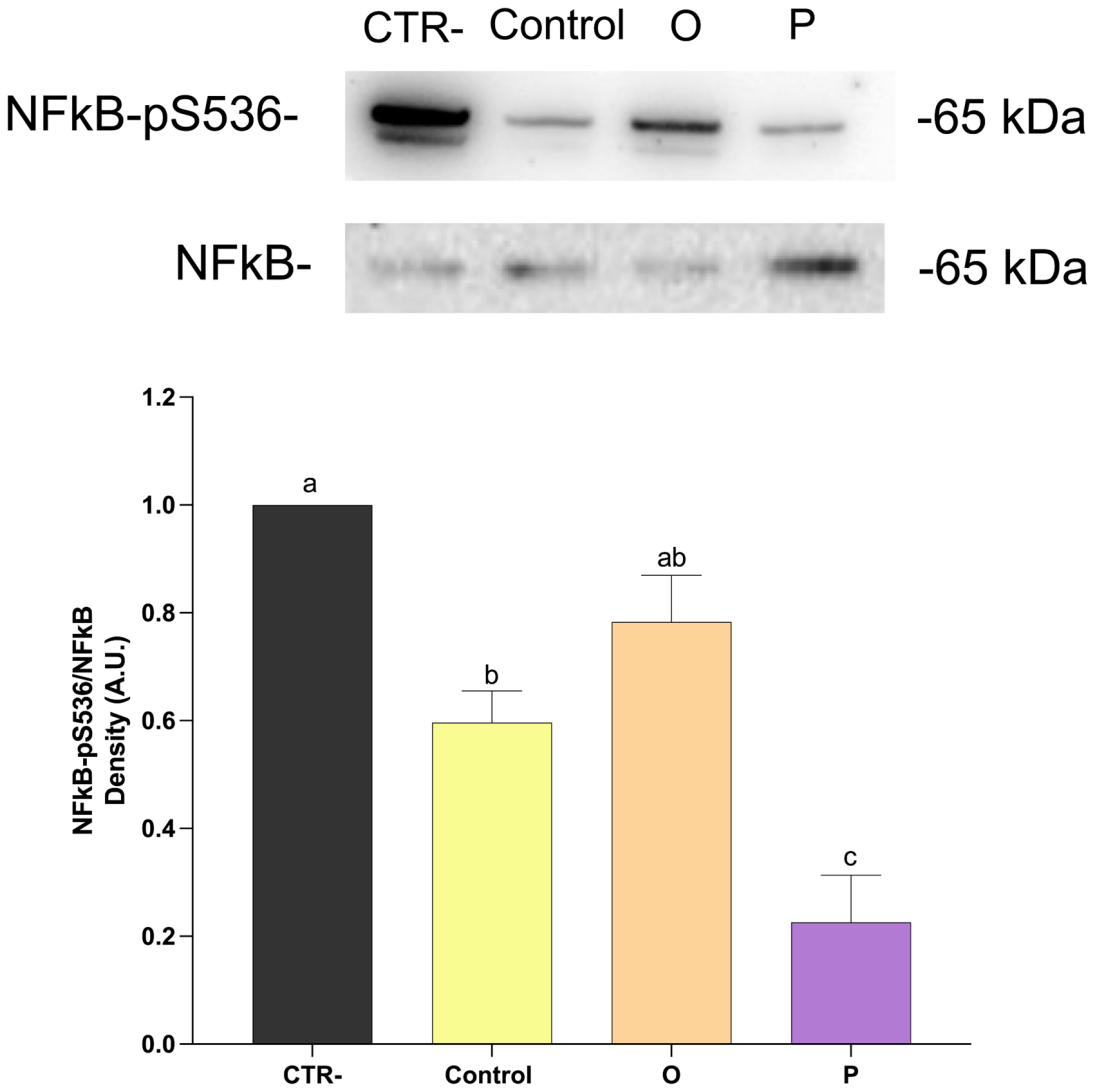
Western blotting analysis of NFκB-pS536 and NFκB proteins in Caco-2 cells treated with digested pasta (CTR-, untreated cells; Control, conventional pasta; O, enriched pasta with orange carrot; P, enriched pasta with purple carrot) and ratio NFκB-pS536/NFκB. Data are expressed as mean ± SEM. Bars with different letters indicate a significant (*p* ≤ 0.05) difference.

**Figure 5:**
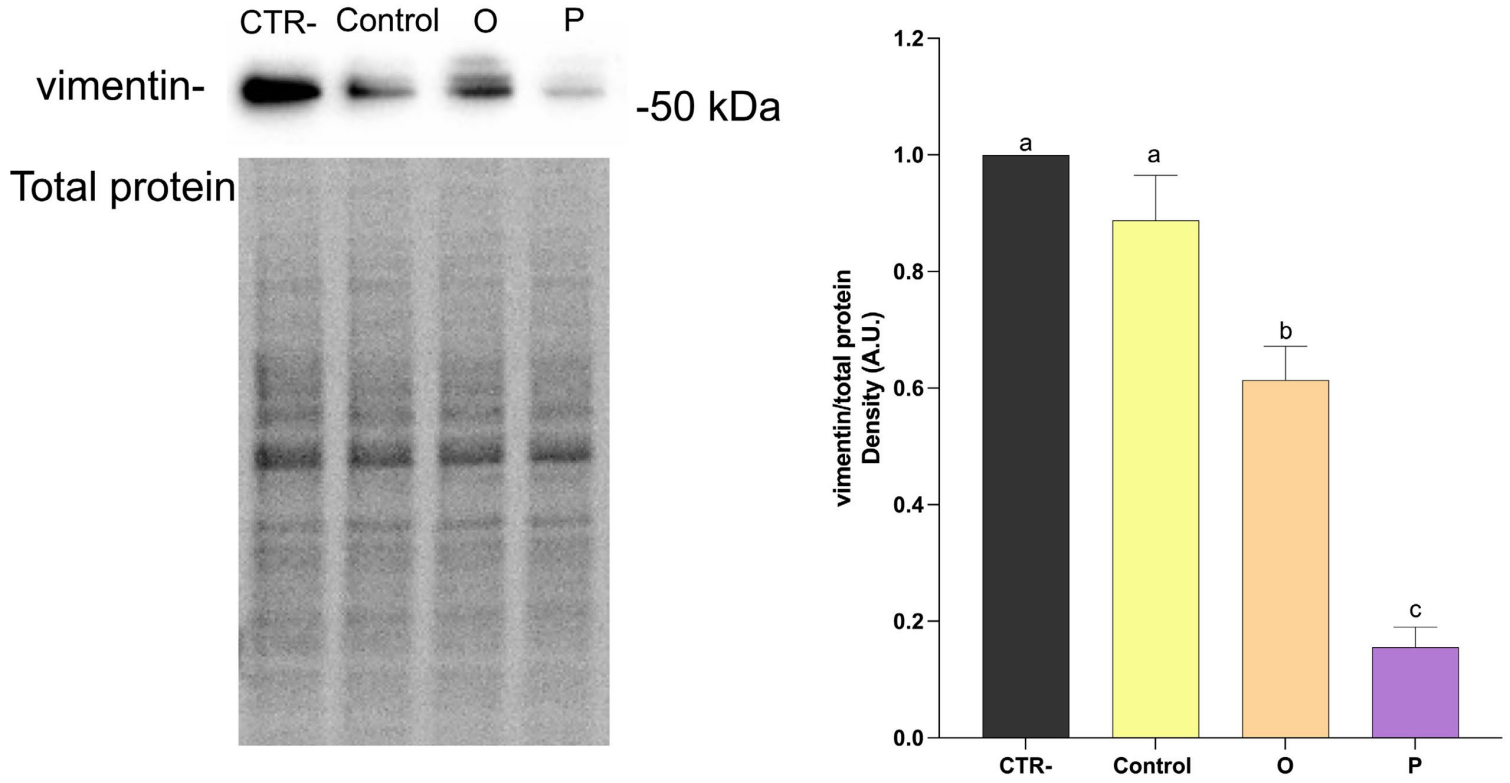
Western blotting analysis of vimentin and total proteins in Caco-2 cells treated with digested pasta (CTR-, untreated cells; Control, conventional pasta; O, enriched pasta with orange carrot; P, enriched pasta with purple carrot) and ratio vimentin/total proteins. Data are expressed as mean ± SEM. Bars with different letters indicate a significant difference (*p* ≤ 0.05).

**Figure 6:**
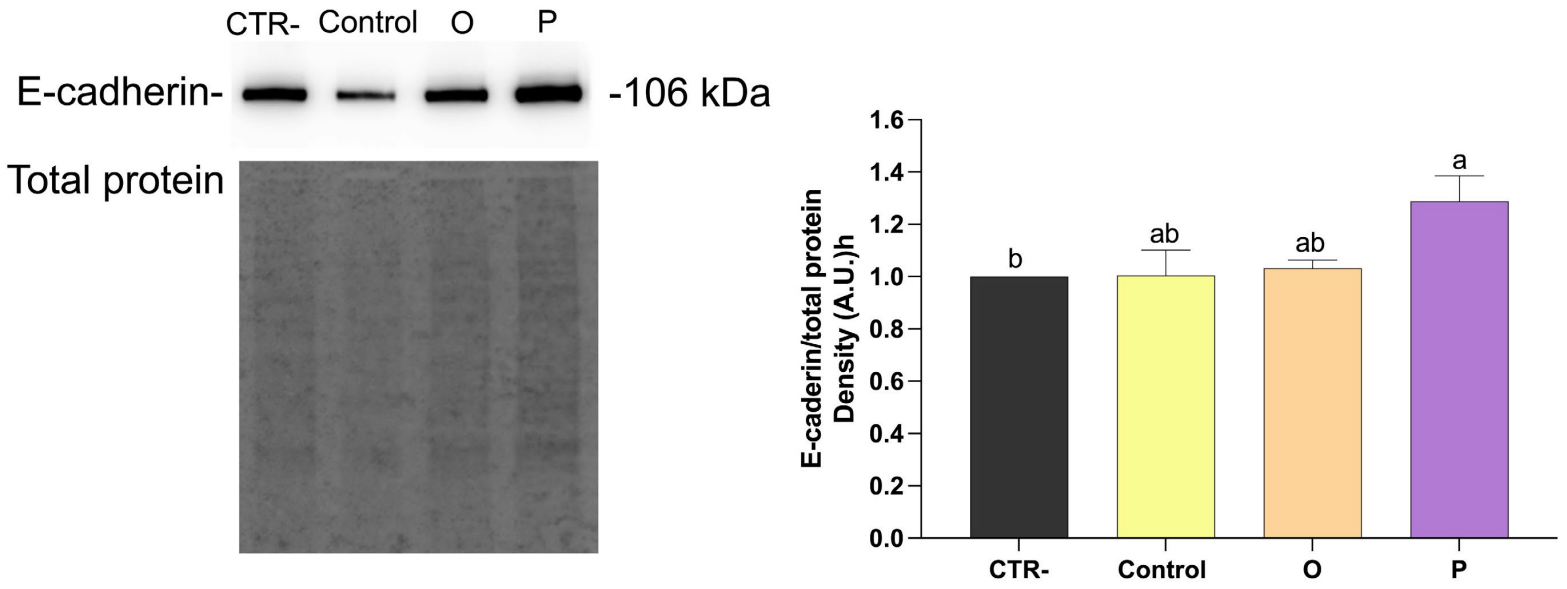
Western blotting analysis of E-cadherin and total proteins in Caco-2 cells treated with digested pasta (CTR-, untreated cells; Control, conventional pasta; O, enriched pasta with orange carrot; P, enriched pasta with purple carrot) and ratio E-cadherin/total proteins. Data are expressed as mean ± SEM. Bars with different letters indicate a significant difference (*p* ≤ 0.05).

**Figure 7:**
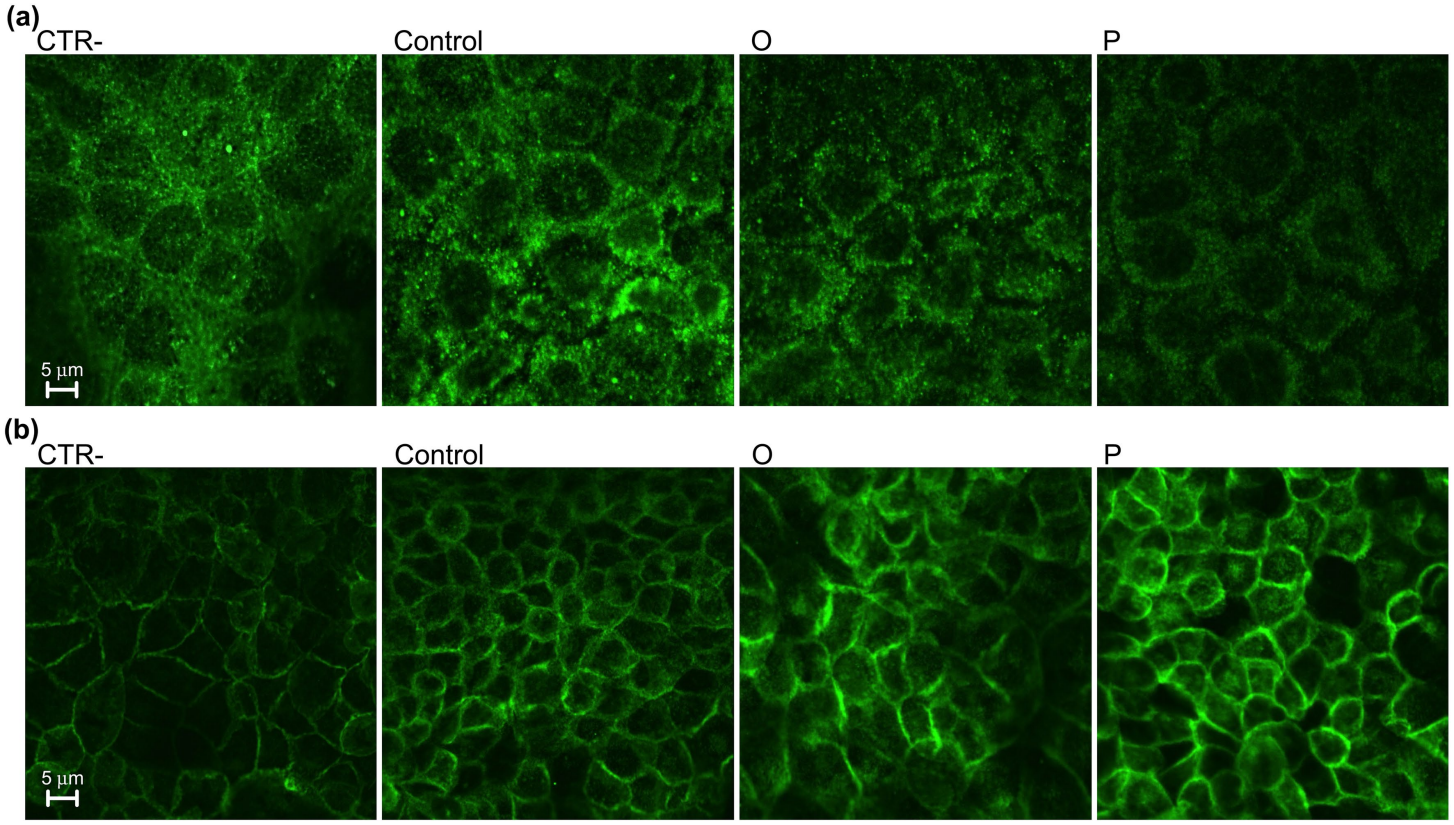
Confocal immunofluorescence visualization of vimentin (panel a) and E-cadherin (panel b) in Caco-2 cells treated with digested (CTR-, untreated cells; Control, conventional pasta; O, enriched pasta with orange carrot; P, enriched pasta with purple carrot).

As shown in Figure 4, treatment of Caco-2 cells with digested control pasta resulted in a significant reduction (*p* ≤ 0.05) of the ratio NFκB-pS536/NFκB, compared to untreated cells (CTR-). No significant differences were found for cells treated with digested O. On the contrary, cells treated with digested P showed the lowest NFκB-pS536/NFκB ratio. Western blotting analysis was carried out also to evaluate the effect of digested pasta on proteins related to epithelial-mesenchimal transition (EMT). As shown in Figure 5, treatment with conventional pasta digest did not cause differences in vimentin expression compared to CTR-. However, a significant (*p* ≤ 0.05) decrease was observed for cells treated with digested O and, especially, P. To further evaluate the effect of the samples on EMT, we carried out Western blotting analysis to assess the level of synthesis of E-cadherin, an adhesion protein present in the adherent junctions of epithelial cells. As shown in Figure 6, cells treated with digested P showed a significant (*p* ≤ 0.05) increase in the E-cadherin/total proteins ratio. Caco-2 cells were subjected to immunofluorescence experiments (Figure 7) that revealed an interesting modulation of the expression and distribution of both vimentin and E-cadherin in response to treatment with digested pasta samples. Vimentin decreased in cells treated with digested O and, especially P compared to untreated cells and cells treated with digested conventional pasta (Figure 7A). Conversely, an increase of the E-cadherin (Figure 7B) was detected in cells treated with digested O and, especially, P.

## 4 Discussion

This study provides a comparative evaluation of fresh pasta enriched with orange or carrot flour and conventional durum wheat pasta, highlighting differences in nutritional profile, sensory and colour characteristics, and *in vitro* biological responses. Replacing 12.5% of durum wheat semolina with orange or purple carrot flour did not negatively impact the shelf life and sensory parameters of fresh pasta. Microbiological analyses confirmed that both enriched pastas (O and P, with orange and purple carrot flour, respectively), stored at 4 °C under modified atmosphere packaging, maintained the same shelf-life (110 days) as the conventional pasta. These results are consistent with previous findings regarding fresh pasta enriched with plant-based ingredients (37, 38).

As regards sensory perception, both the O and P pasta samples were characterized by distinctive but not unpleasant carrot taste and odour, although the panel was too homogeneous in terms of participants’ age. A further limitation consisted in the low number of panellists, of course far from being fully representative of consumers’ perception. Previously Sule and Abu (12) reported that aroma and taste of pasta enriched with 15% carrot flour were enhanced, compared to conventional pasta. Appearance of pasta at 15% of replacement with carrot flour received lower, although more than sufficient, score than 100% wheat-based pasta (13). The same authors reported lower score for colour of enriched pasta, although well above the sufficiency, compared to conventional pasta (13). In the current study, O pasta was more appreciated for its colour than the control, consistent with the higher b* value evaluated by colorimetric analysis. Recently, Luca et al. (39) have reported that the use of pomace from orange cultivars of carrot as non-conventional ingredient (at 12% replacement) of pasta caused decreased brightness and increased red nuance, compared to conventional pasta.

From a nutritional point of view, both enriched pastas contained around 7% of fibre and therefore, according to EC Regulation 1924/2006 (40), could be labelled as “rich in fibre.” This result is in accordance with previous studies (12, 13) which reported that pasta containing 15% of carrot flour had higher fibre than conventional pasta. Being non digestible by humans, dietary fibre plays an important function related to intestinal motility (41). Although it is known that fibre can be fermented by gut microbiota (42), when we simulated digestion and colonic fermentation of pasta, we found no significant effect by the two enriched pasta samples on the cell density of most culturable bacterial groups subjected to analysis. However, the use of one single donor for simulating the microbial community of colon is a limitation of this analysis, because in that way interindividual variability in gut microbiota composition and response is disregarded. In addition, we did not consider eventual variations caused by pasta digestion at metabolites level. Besides fibre, both the enriched pastas showed higher contents of minerals, among which calcium and potassium, consistently with previous studies (12, 13). In the study by Sule and Abu (12), concentration of magnesium in pasta enriched with carrot flour did not differ from the conventional pasta, as in the current study. Differently from our study, they found higher iron but lower zinc in enriched pasta (12). As expected, a notable difference in β-carotene content emerged between the two pastas enriched with carrot flour; in fact, O pasta contained the highest level, consistent with the strong cultivar dependence of carotenoid content (5). Indeed, β-carotene in purple carrots can be up to 10 times lower than in orange ones. We found a concentration of β-carotene in the pasta enriched with orange carrot flour lower than that (3 mg/100 g) reported by Sule and Abu (12), but higher than that (0.047 mg/100 g) of an experimental pasta enriched with 10% carrot flour (15). Because of the high β-carotene content (about 15% of the recommended daily allowance of vitamin A) found for O, the nutrition claim “source of vitamin A (as β-carotene)” could be used for that pasta (Regulation (EU) No 1169/2011) (43). Carrot cultivar could also explain some differences between O and P, in terms of free essential amino acids, such as Trp and valine. Both enriched pastas had lower energy values than the control pasta. Previous studies reported that inclusion of non-conventional vegetables as pasta supplementary ingredients resulted in a decrease of energy value (17, 44). Since carrots are well-known for their antioxidant potential (5), we evaluated the antioxidant activity of pasta enriched with carrot flours, in relation to the content of total phenols and anthocyanins. Although we found that phenols and anthocyanins decreased upon processing of raw material into pasta, mainly due to exposure to high temperature (45, 46), pasta enriched with purple carrot flour (P) showed the highest antioxidant activity. Pasta enriched with orange carrot flour (O) had higher *in vitro* antioxidant activity than control, in accordance with previous research (15). This result is not reflected by the concentration of total phenols, which were higher in the control than in O, but it could be due to high content of β-carotene in O. Indeed, β-carotene, as well as other carotenoids, is known to possess several biological activities, including antioxidant (47).

To verify whether the observed antioxidant properties resulted in a biological response, pasta digests were tested on human intestinal Caco-2 cell line exposed to a synthetic pro-oxidant compound (tBHP). Although no statistically significant reduction in intracellular ROS levels was observed, a slight decreasing trend was noted in cells treated with P pasta digest compared to control. This trend should be interpreted with caution, as it does not conclusively demonstrate an antioxidant effect under the tested conditions. The discrepancy between the results obtained by *in vitro* antioxidant assays and cellular models is common and can be explained by the fundamental differences between these assays. In detail, *in vitro* antioxidant assays measure the direct free radical scavenging capacity of compounds under simplified conditions (ethanolic extract from pasta samples). In contrast, assays with cellular models reflect more complex biological processes, such as the bio-accessibility (influenced by the matrix), cellular uptake, metabolism and intracellular distribution of bioactive compounds (48, 49). In this context, the phenolic compounds and anthocyanins released during simulated digestion may have been insufficiently bioavailable or may have not been efficiently absorbed by Caco-2 cells to exert a measurable intracellular antioxidant effect. The moderate response observed for ROS levels may be due to the variability inherent in the simulated digestion and the relatively low level of semolina substitution with carrot flour. Nevertheless, previous studies have reported similar results, showing a reduction in oxidative stress in Caco-2 cells treated with anthocyanin-rich purple carrot extracts (50). As antioxidant and anti-inflammatory activities are closely related (51), we explored the potential anti-inflammatory effects further by assessing the NFκB signalling pathway, which plays a central role in numerous inflammatory conditions (52, 53). Analysis of the phosphorylation of the p65 subunit of NFκB revealed the lowest levels in cells exposed to P pasta digest, suggesting a potential attenuation of the pro-inflammatory pathway under our experimental conditions. This finding is consistent with previous studies that demonstrated that anthocyanins and other phenolic compounds found in carrots, including chlorogenic and caffeic acids, may modulate NFκB activation not only in intestinal cell models and Caco-2/RAW264.7 co-culture systems, but also in a mouse model (54–56).

NFκB inhibition is often associated with the modulation of the EMT-related processes (57). In the present study, exposure to pasta digest resulted in decreased vimentin levels in both enriched samples. However, a significant increase in E-cadherin was observed only in cells treated with P-pasta digest, as confirmed by immunofluorescence analysis. Combination of vimentin down-regulation and E-cadherin up-regulation is generally considered to be indicative of modulation of markers related to EMT (56, 58, 59). However, these results should be interpreted with caution as they derive from one single *in vitro* model and do not provide sufficient evidence of anti-metastatic effects. These results are consistent with previous studies showing that foods enriched with carrots can retain biologically active phenolic compounds and anthocyanins, even after *in vitro* digestion (60–62). Similarly, anthocyanin extracts from purple carrots have been shown to reduce the expression of pro-inflammatory markers and mediators in Caco-2 cells and RAW264.7 co-culture models (55). As anthocyanins have been shown to have antiproliferative effects on cancer cells (63), it is reasonable to hypothesize that these compounds largely contribute to the observed biological responses in purple carrot pasta. Overall, these results suggest that pasta enriched with purple carrots may preserve bioactive compounds capable of modulating oxidative stress and inflammatory markers *in vitro*. However, these results are based on a single intestinal cell model and should be interpreted with caution, as they may not accurately reflect physiological responses *in vivo*. Further studies using additional models and *in vivo* systems are required to confirm these effects.

## 5 Conclusion

The enrichment of fresh pasta, produced at an industrial level, with carrot flours, particularly from purple carrots, resulted in a product with an improved nutritional profile compared to conventional durum wheat pasta. From a nutritional perspective, carrots-enriched fresh pasta provided high levels of fibre, certain minerals, β-carotene, and bioactive compounds such as phenolics and anthocyanins. These compositional improvements were associated with enhanced antioxidant capacity and anti-inflammatory properties. Notably, *in vitro* experiments on Caco-2 cells supported the biological relevance of these properties, showing inhibition of NFκB activation and modulation of EMT-related markers, particularly in response to P pasta. Future research could expand sensory evaluation through consumer tests and envisage *in vivo* trials aimed to confirm the anti-inflammatory properties of pasta enriched with carrot flour. Overall, these results support the potential of carrot flour as a valuable functional ingredient to produce fresh pasta, contributing to the sustainable valorisation of carrot-derived-by products.

## Data Availability

The original contributions presented in the study are included in the article/Supplementary material, further inquiries can be directed to the corresponding author.
