# Supplementary material for: Carrot flour-enriched pasta with in vitro antioxidant activity and capacity of modulating inflammatory pathways in human colon cell model
Source: Front Nutr. 2026 May 20;13:1818508. doi: 10.3389/fnut.2026.1818508 (PMC13231734; doi:10.3389/fnut.2026.1818508)
Supplement: Supplementary file 1 [file Table_1.docx]

**Supplementary Table 1.** Microbial groups, culture media used, and conditions of incubation for microbiological analyses of faecal batch that contained digested pasta

| **Microbial group** | **Culture medium** | **Incubation conditions (temperature, time, aerobiosis/anaerobiosis)** |
| --- | --- | --- |
| Total aerobic microorganisms | Plate Count agar | 37°C, 48 h, aerobiosis |
| Total anaerobes | Wilkins-Chalgren agar | 37°C, 48 h, anaerobiosis |
| Rod-shaped lactic acid bacteria | de Man, Rogosa, and Sharpe (MRS) agar | 37 °C, 48 h, aerobiosis |
| Coccus-shaped lactic acid bacteria | M17 agar | 37 °C, 48 h, aerobiosis |
| Total coliforms | Violet Red Bile Glucose agar | 37 °C, 24 h, aerobiosis |
| Bifidobacteria | Bifidobacterium agar | 30°C, 48 h, anaerobiosis |
| Clostridia | Reinforce Clostridial Agar (RCA) | 37°C,48 h, anaerobiosis |

**Supplementary Table 2.** Profile of free amino acids (mg/kg) of conventional pasta (control), pasta enriched with orange (O) or purple (P) carrot flour, before (U) and after (C) cooking.

| **mg/kg** | **Control-U** | **O-U** | **P-U** | **Control-C** | **O-C** | **P-C** |
| --- | --- | --- | --- | --- | --- | --- |
| ***Asp*** | 32.02 | 43.75 | 42.43 | 8.78 | 8.66 | 15.03 |
| ***Thr*** | 3.32 | 5.79 | 5.86 | 0.97 | 1.23 | 1.96 |
| ***Ser*** | 4.60 | 7.77 | 5.19 | 1.40 | 1.57 | 2.00 |
| ***Asn*** | 66.01 | 90.21 | 74.93 | 17.77 | 18.58 | 28.41 |
| ***Glu*** | 23.70 | 29.89 | 31.48 | 6.31 | 5.19 | 10.81 |
| ***Gly*** | 6.94 | 6.17 | 4.89 | 1.64 | 1.26 | 1.91 |
| ***Ala*** | 17.56 | 30.78 | 26.56 | 4.49 | 5.75 | 9.77 |
| ***Val*** | 3.60 | 11.54 | 11.24 | 0.88 | 2.34 | 4.33 |
| ***Cys*** | 2.78 | 1.02 | 0.85 | 0.72 | 0.88 | 0.75 |
| ***Ile*** | 2.19 | 3.52 | 3.78 | 0.60 | 0.70 | 1.52 |
| ***Leu*** | 7.27 | 7.19 | 6.88 | 2.77 | 2.09 | 3.06 |
| ***Tyr*** | 4.45 | 3.91 | 3.49 | 1.66 | 0.98 | 1.48 |
| ***Phe*** | 4.00 | 4.79 | 3.97 | 1.28 | 1.10 | 1.76 |
| ***Gaba*** | 19.98 | 31.27 | 17.72 | 5.37 | 6.42 | 6.69 |
| ***Amm*** | 3.10 | 4.33 | 5.41 | 0.87 | 1.01 | 2.55 |
| ***Lys*** | 5.02 | 4.51 | 4.00 | 1.59 | 1.25 | 1.76 |
| ***His*** | 1.57 | 2.10 | 2.06 | 0.51 | 0.53 | 1.08 |
| ***Trp*** | 25.96 | 22.37 | 20.61 | 8.31 | 6.24 | 8.50 |
| ***Arg*** | 9.18 | 11.95 | 19.44 | 2.92 | 3.46 | 9.04 |
| ***Pro*** | 4.24 | 4.67 | 3.06 | 0.99 | 0.70 | 1.16 |

**Supplementary Table 3.** Cell densities of total mesophilic aerobic bacteria (TMA), presumptive lactic acid bacteria (LAB), and presumptive yeasts found in conventional pasta (control), pasta enriched with orange (O) or purple (P) carrot flour on the day of production (T0), and after 30 (T30), 60 (T60), 90 (T90) and 110 (T110) days of refrigerated storage.

|  | **Pasta** | **TMA** | **LAB** | **Yeasts** |
| --- | --- | --- | --- | --- |
| **T0** | **Control** | 1.78±0.00 | n.d.^*^ | n.d. |
|  | **O** | 1.65±0.92 | 2.04±0.06 | n.d. |
|  | **P** | 3.42±0.17 | 1.60±0.00 | n.d. |
| **T30** | **Control** | 2.50±0.03 | 2.15±0.21 | 2.29±0.02 |
|  | **O** | 3.61±0.02 | 2.89±0.01 | 2.75±0.21 |
|  | **P** | 3.71±0.01 | 2.70±0.06 | 2.87±0.05 |
| **T60** | **Control** | n.d. | n.d. | n.d. |
|  | **O** | 2.54±0.09 | n.d. | n.d. |
|  | **P** | 2.32±0.03 | n.d. | n.d. |
| **T90** | **Control** | 2.66±0.05 | n.d. | n.d. |
|  | **O** | 3.02±0.09 | 2.4±0.13 | n.d. |
|  | **P** | 3.2±0.28 | 2.62±0.45 | n.d. |
| **T110** | **Control** | 2.95±0.00 | n.d. | n.d. |
|  | **O** | 4.36±0.42 | n.d. | n.d. |
|  | **P** | 3.02±0.03 | n.d. | n.d. |

Data are expressed as mean ± SD. * n.d., below the detection limit

**Supplementary Table 4.** Cell densities of faecal microbiota after simulated colonic fermentation of the conventional pasta (Control), pasta enriched with orange (O) or purple (P) carrot flour.

|  | Total aerobic microorganisms | Rod-shaped lactic acid bacteria | Total coliforms | Total anaerobes | Coccus-shaped lactic acid bacteria | Clostridia | Bifidobacteria |
| --- | --- | --- | --- | --- | --- | --- | --- |
| Control | 8.9 ±1.0^a^ | 6.1 ±0.1^a^ | 5.9 ± 0.7^a^ | 8.8 ± 0.9^a^ | 7.1 ±0.2^a^ | 8.8 ±0.2^a^ | 3.2 ±0.2^a^ |
| O | 8.6 ±0.5^a^ | 6.0 ±0.2^ab^ | 6.1 ±0.7^a^ | 8.9 ±0.8^a^ | 7.0 ±0.1^a^ | 6.6 ±0.6^c^ | 3.3 ±0.5^a^ |
| P | 9.1 ±1.1^a^ | 5.7±0.2^b^ | 5.6 ±0.7^a^ | 9.2±1.2^a^ | 6.4 ±0.1^b^ | 8.0±0.01^b^ | 3.2 ±0.2^a^ |

Data are expressed as mean ± SD. In the same column different superscript letters differ significantly with *p* ≤ 0.05.

**Supplementary Table 5.** Viability of cells treated for 24 hours with digested samples of conventional pasta (Control), pasta enriched with orange (O) or purple (P) carrot flour and differently diluted. Untreated cells were used as negative control (CTR-).

| ***Dilution of digested pasta*** | **CTR-** | **Control** | **O** | **P** |
| --- | --- | --- | --- | --- |
| ***1:200*** | 1 ± 0 ^a^ | 1.00 ± 0.07 ^a^ | 0.94 ± 0.06 ^a^ | 0.97 ± 0.06 ^a^ |
| ***1:150*** | 1 ± 0 ^a^ | 0.99± 0.06 ^a^ | 0.78 ± 0.04 ^b^ | 0.95 ± 0.08 ^a^ |
| ***1:100*** | 1 ± 0 ^a^ | 0.97 ± 0.05 ^a^ | 0.95 ± 0.07 ^a^ | 0.86 ± 0.08 ^a^ |
| ***1:50*** | 1 ± 0 ^a^ | 0.95 ± 0.08 ^ab^ | 0.85 ± 0.08 ^b^ | 0.88 ± 0.02 ^ab^ |

Data are expressed as mean ± SD. In the same row different superscript letters differ significantly with *p* ≤ 0.05.
